# The Impact of Next‐Generation Sequencing on Interobserver Agreement and Diagnostic Accuracy of Deep Penetrating Melanocytic Neoplasms

**DOI:** 10.1111/cup.70049

**Published:** 2025-12-23

**Authors:** Julia Edwin Jeyakumar, Afua Konadu Addo, Haya Mary Beydoun, Shantel Olivares, Armita Bahrami, Thiagarajah Balamurugan, Raymond L. Barnhill, Willeke A. M. Blokx, Klaus J. Busam, Lorenzo Cerroni, Martin Cook, Arnaud de La Fouchardière, Lyn M. Duncan, David E. Elder, Peter Ferguson, Gerardo Ferrara, Iva Johansson, Jennifer S. Ko, Ji Eun Kwon, Gilles Landman, Cecilia Lezcano, Lori Lowe, Daniela Massi, Jane Messina, Daniela Mihic‐Probst, Douglas C. Parker, Margaret Redpath, Michael R. Sargen, Richard A. Scolyer, Christopher R. Shea, Michael Tetzlaff, Carlos Torres‐Cabala, Victor Tron, Xiaowei Xu, Iwei Yeh, Sook Jung Yun, Artur Zembowicz, Pedram Gerami

**Affiliations:** ^1^ Department of Dermatology Feinberg School of Medicine, Northwestern University Chicago Illinois USA; ^2^ Department of Pathology Emory University School of Medicine Atlanta Georgia USA; ^3^ Department of Pathology Royal Surrey NHS Foundation Trust Guildford UK; ^4^ Department of Translational Research Institut Curie, Paris Sciences and Lettres Research University, and UFR of Medicine, University of Paris Cité Paris France; ^5^ Department of Pathology, Division Laboratories Pharmacy and Biomedical Genetics University Medical Center Utrecht Utrecht the Netherlands; ^6^ Department of Pathology Dermatopathology Service, Memorial Sloan Kettering Cancer Center New York City New York USA; ^7^ Department of Dermatology Medical University of Graz Graz Austria; ^8^ Department of Biopathology Centre Leon Bernard Lyon France; ^9^ Dermatopathology Unit, Pathology Service, Massachusetts General Hospital, Harvard Medical School Boston Massachusetts USA; ^10^ Department of Pathology and Laboratory Medicine Division of Anatomic Pathology, Hospital of the University of Pennsylvania Philadelphia Pennsylvania USA; ^11^ Melanoma Institute Australia, The University of Sydney Sydney Australia; ^12^ Department of Tissue and Diagnostic Oncology Royal Prince Alfred Hospital Sydney Australia; ^13^ Anatomic Pathology and Cytopathology Unit, Istituto Nazionale Tumori IRCCS Fondazione 'G. Pascale' Naples Italy; ^14^ Department of Pathology Institute of Biomedicine, Sahlgrenska University Hospital Gothenburg Sweden; ^15^ Department of Anatomic Pathology Cleveland Clinic Cleveland Ohio USA; ^16^ Department of Pathology Ajou University School of Medicine Suwon South Korea; ^17^ Department of Pathology Escola Paulista de Medicina, Universidade Federal de São Paulo São Paulo Brazil; ^18^ Departments of Dermatology and Pathology University of Michigan Medical School Ann Arbor Michigan USA; ^19^ Department of Health Sciences, Section of Anatomic Pathology University of Florence Florence Italy; ^20^ Departments of Pathology and Cutaneous Oncology Moffitt Cancer Center Tampa Florida USA; ^21^ Institute for Pathology and Molecular Pathology, University Hospital Zurich Zurich Switzerland; ^22^ Departments of Pathology and Dermatology Emory University School of Medicine Atlanta Georgia USA; ^23^ Department of Pathology McGill University Quebec Canada; ^24^ Division of Cancer Epidemiology and Genetics National Cancer Institute, National Institutes of Health Rockville Maryland USA; ^25^ Tissue Pathology and Diagnostic Oncology, Royal Prince Alfred Hospital, and NSW Health Pathology Sydney New South Wales Australia; ^26^ Faculty of Medicine and Health, the University of Sydney Sydney New South Wales Australia; ^27^ Charles Perkins Centre, The University of Sydney Sydney New South Wales Australia; ^28^ Department of Medicine Section of Dermatology, University of Chicago Chicago Illinois USA; ^29^ Dermatopathology and Oral Pathology Unit, Departments of Pathology and Dermatology University of California San Francisco California USA; ^30^ Departments of Pathology and Dermatology The University of Texas MD Anderson Cancer Center Houston Texas USA; ^31^ Lifelabs and Department of Laboratory Medicine and Pathobiology University of Toronto Toronto Ontario Canada; ^32^ Departments of Pathology and Dermatology University of Pennsylvania Philadelphia Pennsylvania USA; ^33^ Departments of Dermatology and Pathology University of California San Francisco California USA; ^34^ Department of Dermatology Chonnam National University Medical School Gwangju Korea; ^35^ Dermatopathology Consultations LLC Lahey Clinic and Tufts Medical School Boston Massachusetts USA

**Keywords:** deep penetrating melanocytic tumor of uncertain malignant potential, deep penetrating nevus, deep penetrating nevus‐like melanoma, plexiform melanoma, WNT‐activated Melanocytoma

## Abstract

**Background:**

Next‐generation sequencing (NGS) is becoming more commonly used for diagnosis in dermatopathology. It's critical to appraise its efficacy and limitations. Distinguishing benign deep penetrating nevi (DPN) from deep penetrating like‐melanoma (DPN‐M) is a challenging diagnostic scenario even for experienced dermatopathologists.

**Methods:**

We sent a two‐phase survey (pre‐and postgenomics) to 32 experienced dermatopathologists to evaluate 39 diagnostically challenging cases from the DPN/WNT‐activated family of melanocytic neoplasms.

**Results:**

With NGS data, interobserver agreement improved from 0.41 to 0.51 (*p* < 0.0001) in distinguishing DPN‐M from nonmelanoma cases. Overall diagnostic accuracy improved, mostly driven by a 16% increase in accurate diagnosis of DPN‐M. However, in two cases, the inclusion of genomics shifted the majority vote from a correct to an incorrect diagnosis. A total of 218 diagnostic changes occurred between Survey 1 and 2. Among the changes, 132 votes moved toward the correct diagnosis while 86 moved toward an incorrect diagnosis. The shift in voting which resulted in improved diagnostic accuracy was statistically significant (*p* = 0.0001).

**Conclusions:**

NGS has the potential to improve interobserver agreement and diagnostic accuracy. We provide guidance on the utilization of bioinformatic data to maximize its benefits and improve diagnostic accuracy and interobserver agreement.

## 1 Introduction

Recent studies have identified genomic differences between DPN (deep penetrating nevi) and DPN‐M (deep penetrating nevus like melanoma) [1, 2] suggesting next‐generation sequencing (NGS) could be utilized to aid in diagnosis. Both tumors typically carry mutations involving the WNT pathway. DPN‐M, on average, have higher tumor mutational burden (TMB) and more pathogenic variants. Pathogenic variants in *TERT‐*promoter and *CDKN2A* are common in DPN‐M but not in DPN. Likewise, pathogenic variants involving the SWItch/Sucrose NonFermentable (SWI/SNF) complex (*ARID1A*, *ARID1B*, *ARID2*, *PBRM1*, *SMARCA4*) are significantly more frequent in DPN‐M compared to DPNs (7/11, 64% vs. 2/24, 8%; *p* = 0.02) [1].

In this study, we provided an expert panel of melanoma pathologists a set of cases with morphologic features typical of the WNT‐activated family of melanocytic neoplasms. We asked for their diagnostic impression before and after receiving the genomic profile of the tumor and assessed changes in interobserver agreement and diagnostic accuracy.

## 2 Materials and Methods

### 2.1 Case Selection

The study was carried out with the approval of our Institutional Review Board (STU0001127). Our dermatopathology database was queried from 2020 to 2024 for the following diagnoses: DPN, Deep penetrating melanocytic tumor of uncertain malignant potential (DPMT‐UMP), and DPN‐M. We identified 340 cases. Cases without NGS data or sufficient tissue to perform NGS studies were excluded, yielding 39 cases.

### 2.2 Genomic Sequencing and Immunohistochemistry

NGS was conducted for all 39 cases either as part of the clinical diagnostic workup or for research purposes. All cases were tested on either the PGDx or Tempus xT/xO panel [3, 4, 5, 6, 7, 8].

Immunohistochemistry (IHC) staining results were available for 27 cases and included: HMB‐45 (*n* = 1), Ki‐67 (*n* = 6), MART‐1 (*n* = 6), SOX‐10 (*n* = 9), S‐100, p16 (*n* = 20), BRAF V600E (*n* = 6), PRAME (*n* = 19), ALK (*n* = 1), BAP1 (*n* = 5), NRAS Q61R (*n* = 2), and β‐catenin (*n* = 14). β‐catenin IHC was interpreted as positive by nuclear positivity throughout the clonal population of melanocytes [9].

### 2.3 Survey Administration and Consensus Diagnosis

We distributed two surveys containing all 39 de‐identified cases to 32 expert dermatopathologists from the International Melanoma Pathology Study Group (IMPSG). Survey 1 contained relevant clinical information (age, sex, and site), any IHC results, and an electronic link to the hematoxylin‐and‐eosin (H&E) slide. Participants were asked to select only one of five diagnoses for each case: (1) Conventional nevus/not DPN pathway, (2) Malignant melanoma/not DPN pathway, (3) DPN, (4) DPMT‐UMP, and (5) DPN‐M. Uncertain malignant potential (UMP) was defined as inconclusive for melanoma based on available data. After completing Survey 1, Survey 2 was provided which contained all information in Survey 1 and genomic data (Table S1). Case order was randomized in each survey.

Consensus diagnosis was established at an in‐person meeting with nine IMPSG members involved in the study. The final consensus diagnosis involved review of clinical information, IHC, histology, and genomics for each case. All members assessed the cases first independently and then in a group setting where the voting took place. Consensus diagnosis was reached when greater than 66% (2/3) of members agreed on a diagnosis. A consensus diagnosis of either DPN, DPMT‐UMP, or DPN‐M was reached for all 39 cases which was used for the gold standard in calculating diagnostic accuracy.

### 2.4 Statistical Analysis

Statistical analysis was done using IBM SPSS Version 29 (IBM Corp, Armonk, NY). Interobserver agreement was calculated using the Fleiss multirater κ test. “A score of ≤ 0 indicates no agreement; 0.01–0.20 slight agreement; 0.21–0.40 fair agreement; 0.41–0.60 moderate agreement; 0.61–0.80 substantial agreement; 0.81–0.99 near perfect agreement; and 1.0 perfect agreement” [10]. Standard error and confidence intervals were calculated for Fleiss' Kappa. *Z*‐test was utilized to assess the change in Fleiss' Kappa between Survey 1 and 2.

McNemar *χ* ^2^ test was performed to assess any statistically significant differences between the two surveys. Diagnostic accuracy was calculated by comparing each voter's diagnosis to the consensus diagnoses for each case. Percentage agreement was calculated by dividing the most frequently selected diagnostic score by the total number of votes in Survey 1 and Survey 2.

## 3 Results

### 3.1 General and Clinical Features

Among 39 cases, consensus review of all data including genomics by an expert panel favored a diagnosis of DPN in 21 cases, DPMT‐UMP in seven cases and DPN‐M in 11 cases. Among 189 votes for the 21 DPN cases, 167 votes were for DPN (88%), 21 for DPN‐UMP (11%), and 1 for DPN‐M (< 1%). Among 99 votes for 11 DPN‐M cases, 95 were for DPN‐M (96%) and four for DPN‐UMP (4%). Among 63 votes for seven DPN‐UMP cases, 43 were for DPN‐UMP (75%), 14 for DPN and six for DPN‐M. The DPN‐M cases occurred in older patients, but there was no statistical significance in comparing age, gender or body site distribution for the DPN‐M and DPN/DPMT‐UMP cohorts (Table 1).

**TABLE 1:** Clinical features of DPN‐M and DPMT‐UMP/DPN cases.

Characteristics	DPN‐M	DPMT‐UMP/DPN	*p*
*N*	11	28	
Mean age years (range)	49 (23–74)	41(10–83)	0.22
Female:Male	9:2	17:11	0.21
Location			
Head and neck	18% (2)	36% (10)	0.39
Trunk	55% (6)	32% (9)	
Extremities	27% (3)	32% (9)	

*Note*: Statistical significance *p* < 0.05.

Abbreviations: DPN = Deep penetrating nevi; DPMT‐UMP = deep penetrating melanocytic tumor of uncertain malignant potential; DPN‐M = DPN like melanoma.

Clinical follow‐up was available for 8 of 11 DPN‐M cases (73%). Mean follow‐up time was 18 months, ranging from 1 to 38 months. Sentinel lymph node biopsy (SLNB) information was available for seven of eight DPN‐M cases, with four being positive. Two patients developed clinically bulky metastasis to lymph nodes. The remaining six patients had no evidence of recurrence (NER). Follow‐up information was obtained in 15 of 28 nonmelanoma cases (54%) and there were no recurrences (mean of 10 months, ranging from 3 to 20 months).

### 3.2 Genomic Overview of NonMelanoma Cases

**FIGURE 1:**
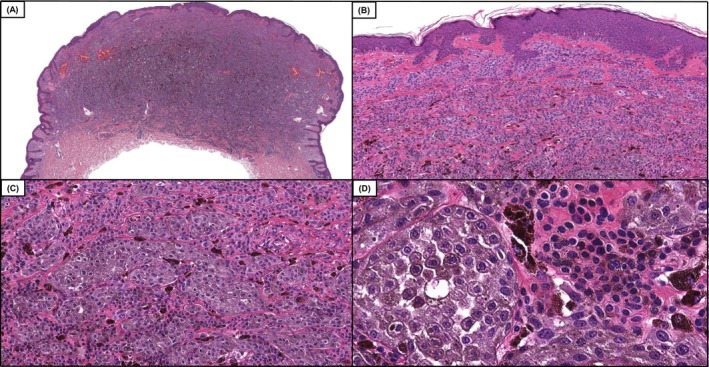
Case 12, a DPN (deep penetrating nevus), in which diagnostic accuracy improved by 13%, following the inclusion of genomic data which included the following pathogenic variants *BRAF* V600E and *CTNNB1* S45F. Low power magnification demonstrates a compound dome‐shaped, phenotypically heterogeneous melanocytic neoplasm. (A and B, H&E, 50× and 100×) On higher power magnification one can appreciate expansile nests of larger epithelioid melanocytes with enlarged nuclei and dusty gray cytoplasmic melanin pigmentation in the cytoplasm adjacent to smaller appearing conventional melanocytes (C and D, H&E, 200× and 400×). The tumor had good overall symmetry, a mitotic count of 1/mm^2^, and lacked necrosis or areas of high‐grade nuclear atypia.

Among 28 nonmelanoma cases, 23 harbored a mutation(s) in the WNT pathway (Figure 1). This involved *CTNNB1* in 19 cases, *APC* in five cases, *CTNNB1* and *APC* in one case. Five cases had neither *APC* nor *CTNNB1* but were included because the morphology was characteristic of DPN. The favored diagnosis in all five was DPN. The driver mutations involved were *BRAF* V600E (*n* = 14), *NRAS* (*n* = 2), *HRAS* (*n* = 1), *KRAS* (*n* = 1), *MAP2K1* alterations (*n* = 6), and unknown (*n* = 4) (Table S1). Pathogenic variants in the SWI/SNF complex were rarely seen in DPN/DPMT‐UMP cases with the following exceptions: *ARID1A* (*n* = 1) and *SMARCA4* (*n* = 1). No cases had pathogenic variants in *TERT*‐promoter or *CDKN2A*. The average TMB was 8.2 m/MB ranging from 0.8 to 20.8 m/MB.

### 3.3 IHC Results of NonMelanoma Group

Among 28 DPN/DPMT‐UMP cases, 19 had IHC. Positive nuclear staining for β‐catenin was seen in 11 of 13 cases (85%). PRAME was negative in 11 of 12 (92%). One DPMT‐UMP case had positive PRAME staining in greater than 75% of cells. P16 staining showed patchy loss in 8 of 14 cases (57%), complete loss in 2 of 14 cases (14%), and retained expression in 4 of 14 cases (29%). β‐catenin IHC was performed in four DPN/DPMT‐UMP cases which did not harbor a pathogenic mutation in the WNT pathway, and three were positive.

### 3.4 Genomic Overview of Melanoma Cases

**FIGURE 2:**
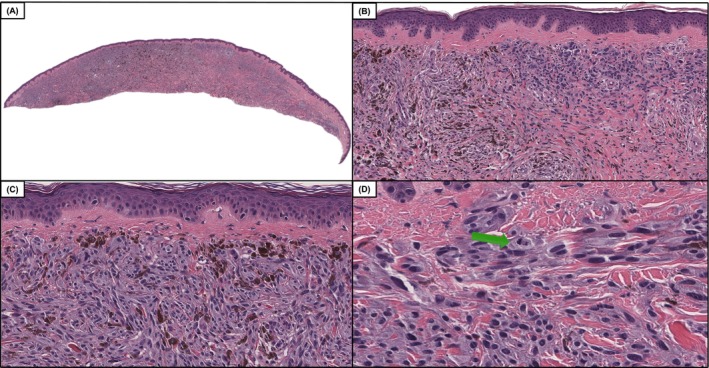
Case 34, a DPN‐M (deep penetrating nevus like melanoma) case with a genomic profile including *TERT* promoter c.‐146C>T, *BRAF* V600E, *CTNNB1* S45F, *CDKN2A* in which saw there was a 66% improvement in diagnostic accuracy following the inclusion of genomics. Low power demonstrates an intradermal melanocytic neoplasm with variable cellular density and notable phenotypic heterogeneity (A and B, H&E, 50× and 100×). Intermediate power demonstrates an area of increased cellularity with markedly atypical melanocytes with pigmented cytoplasm (C, H&E, 200×). Higher power magnification demonstrates indistinct cytoplasmic borders, hyperchromatic and pleomorphic nuclei, and mitotic activity denoted by the green arrow (D, H&E 400×).

The majority of cases (10 of 11, 91%) in the DPN‐M cohort harbored either *CTNNB1* (*n* = 6) or *APC* (*n* = 4) pathogenic variants (Figure 2). One case (32) did not contain *CTNNB1* or *APC* but was included because the histology was typical of the DPN family. Driver mutations involved *BRAF* in 6 of 11 cases (55%), *NRAS* in 3 of 11 cases (27%), *HRAS* in 1 of 11 cases (9%), and unknown in 1 of 11 (9%). Additional pathogenic variants identified in the malignant cohort include: *TERT‐p* (*n* = 7, 64%), *CDKN2A* (*n* = 4, 36%), *TP53* (*n* = 1, 9%), *NF1* (*n* = 2, 18%). Pathogenic variants among the SWI/SNF complex included *ARID1A* (*n* = 1), *ARID1B* (*n* = 2), *ARID2* (*n* = 1), and *SMARCA4* (*n* = 2). Genes with pathogenic alterations occurring more than once in the DPN‐M cohort included: *AR, DNMT3A, ERBB4, GRIN2A*, and *SETD2*. TMB was available for 10 of the 11 DPN‐M, with a mean of 37.1 m/MB and ranged from 6.9 to 160.8 m/MB. This was significantly greater than the nonmelanoma cohort (*p* < 0.05).

### 3.5 IHC Results of Melanoma Group

Eight DPN‐M cases had IHC. PRAME staining was positive in 3 of 7 cases (43%). P16 staining showed complete loss in three of six cases (50%), patchy loss in two of six cases (33%), and retained expression in one case (17%). β‐catenin IHC was performed in one DPN‐M case which did not harbor a pathogenic mutation in the WNT pathway and was negative

### 3.6 Voting Results

**FIGURE 3:**
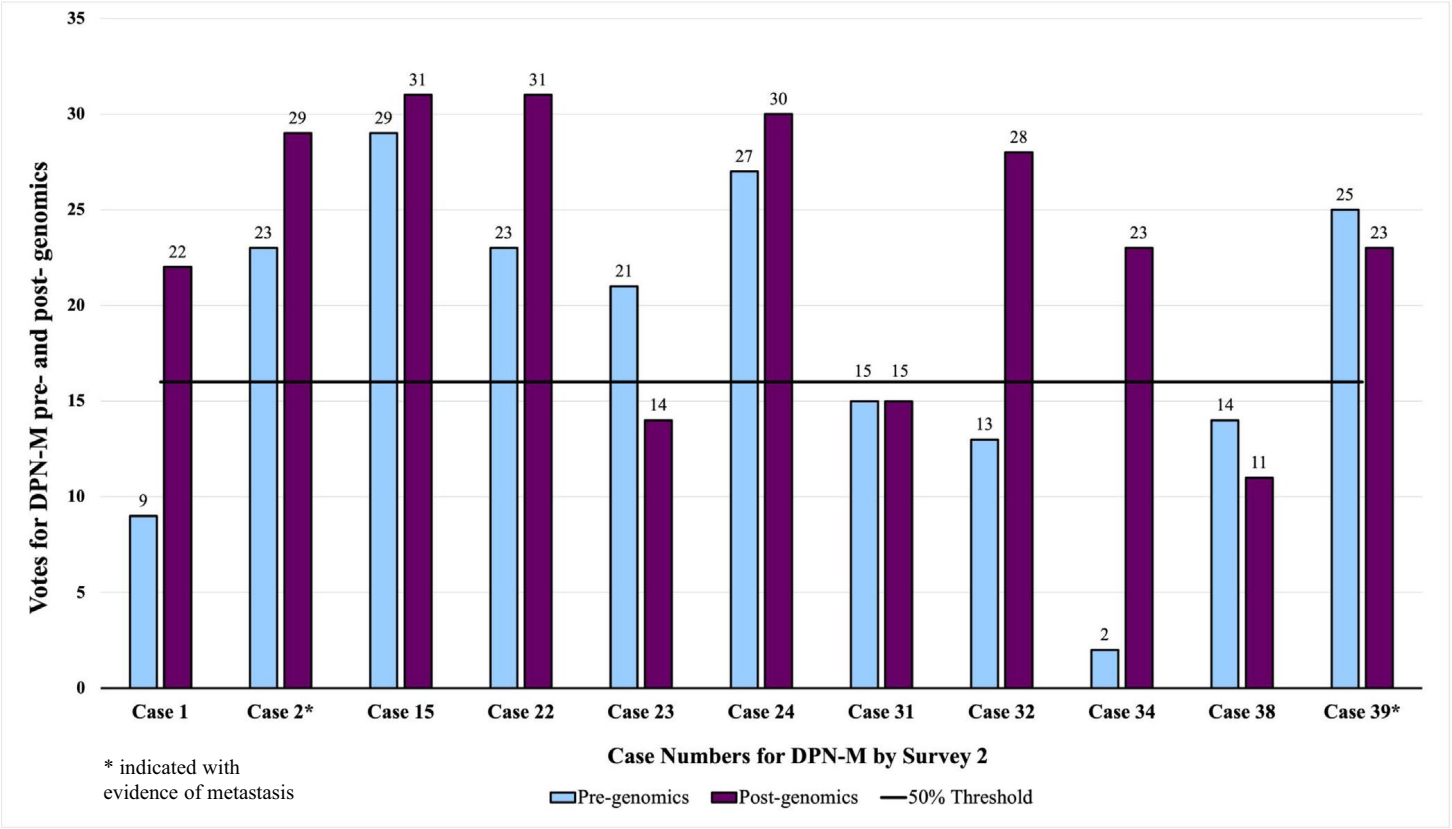
Votes for DPN‐M (deep penetrating nevus like melanoma) among 11 DPN‐M cases before and after assessing genomic information. Cases with asterisks (2, 39) had confirmed metastasis. All cases with at least 50% (≥ 16) of the votes (above the solid line) indicate a majority diagnosis of DPN‐M. All cases with less than 50% (< 16) of the votes (below the solid line) indicate a majority diagnosis of nonmelanoma.

**FIGURE 4:**
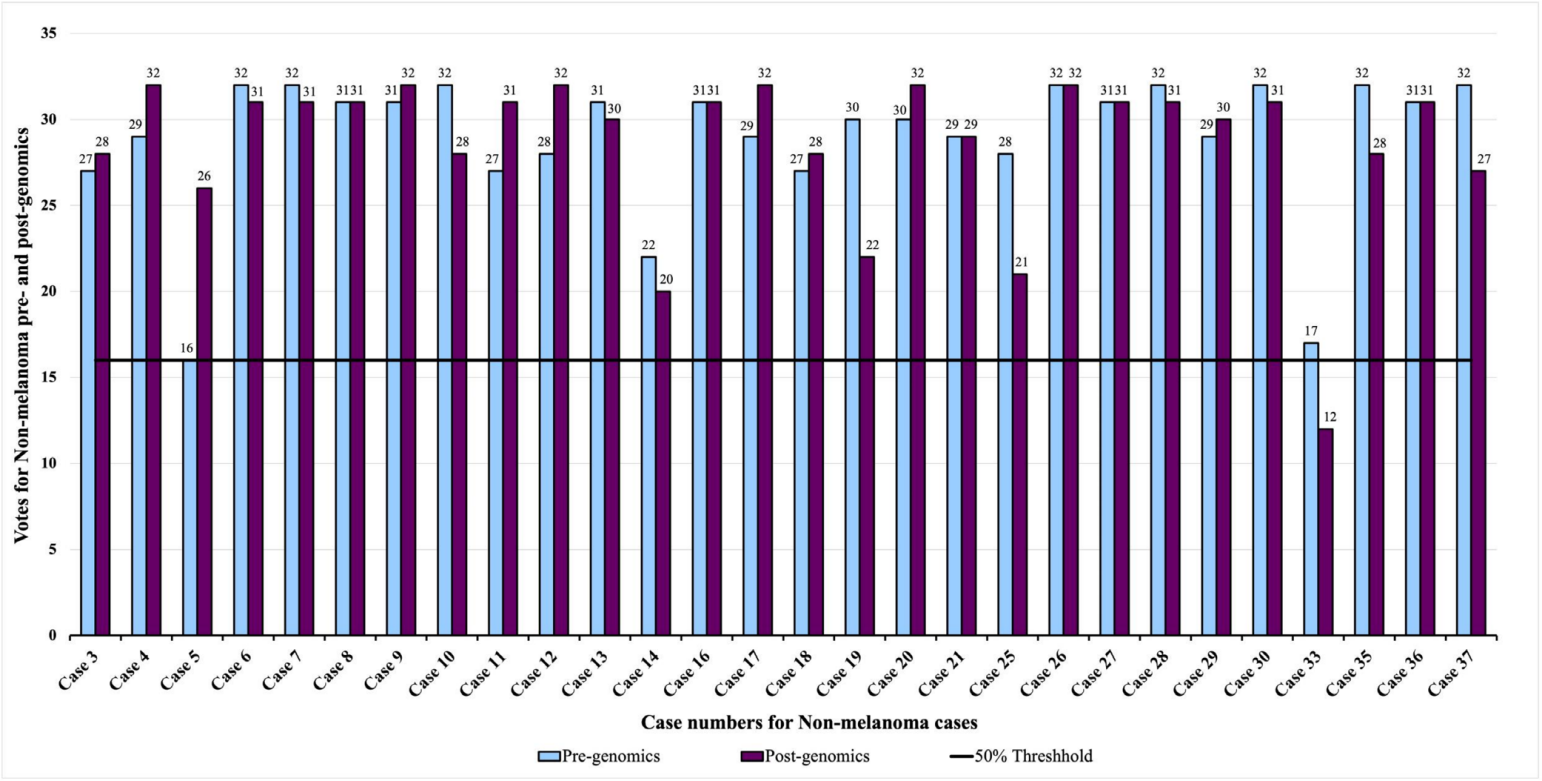
Votes for DPN/DPMT‐UMP (deep penetrating nevi/deep penetrating melanocytic tumor of uncertain malignant potential) among 28 DPN/DPMT‐UMP cases before and after assessing genomic information. All cases with at least 50% (≥ 16) of the votes (above the solid line) indicate a majority diagnosis of nonmelanoma. All cases with less than 50% (< 16) of the votes (below the solid line) indicate a majority diagnosis of DPN‐M (deep penetrating nevus like melanoma).

Interobserver agreement regarding designation of a case as DPN‐M versus DPN/DPMT‐UMP improved from 0.41 to 0.51 (moderate agreement) after genomics. Standard error on the pregenomics Fleiss' Kappa was 0.007 with a confidence interval of 0.391 to 0.419. The postgenomics Fleiss' Kappa had a standard error of 0.007 with a confidence interval of 0.495 to 0.524. The *p*‐value for the *z*‐test comparing the pre and postgenomics Fleiss' Kappa was < 0.0001 indicating the improvement was not random. Among the DPN‐M cases, percentage agreement increased from 57% to 73% after genomics and decreased from 90% to 89% in the DPN/DPMT‐UMP cohort (Figures 3 and 4). There were only two cases in which the majority of votes switched from being aligned with the expert consensus diagnosis before genomics to against the expert consensus diagnosis after genomics.

### 3.7 Diagnostic Accuracy

**FIGURE 5:**
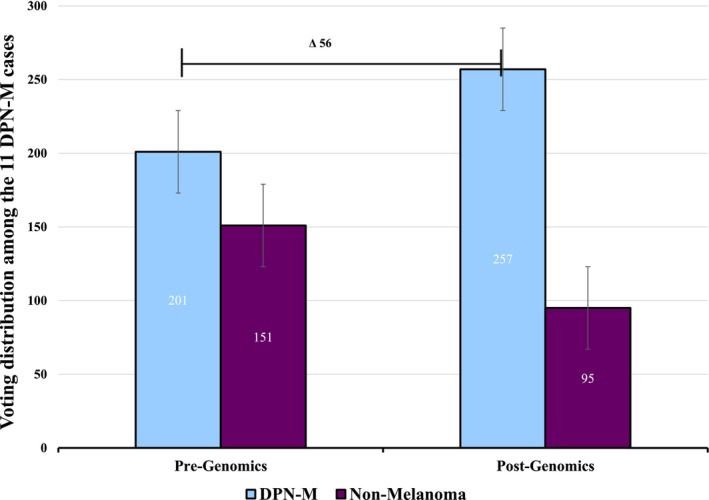
Voting distribution among the 11 DPN‐M (deep penetrating nevus like melanoma) cases demonstrates a 16% improvement in diagnostic accuracy after providing genomic data.

**FIGURE 6:**
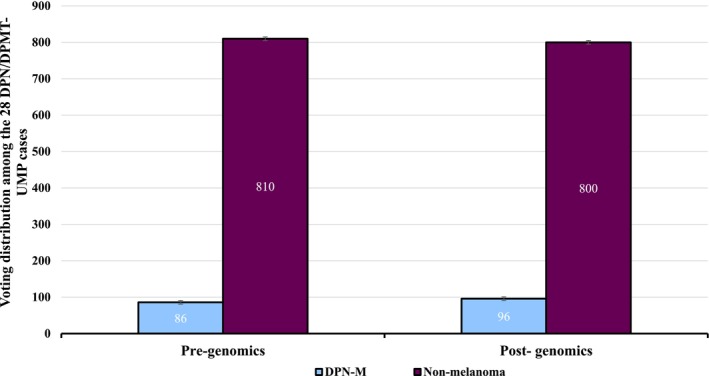
Voting distribution among the 28 DPN/DPMT‐UMP (deep penetrating nevi/deep penetrating melanocytic tumor of uncertain malignant potential) cases demonstrates a 1% decrease in diagnostic accuracy after providing genomic data.

Without genomic data, a total of 1011/1248 votes from 32 participants were aligned with the consensus diagnosis of DPN‐M or nonmelanoma. After genomics, 1057/1248 votes from 32 participants were aligned with the consensus diagnosis. Specifically, 132 votes moved toward the consensus diagnosis while 86 votes moved away from the consensus diagnosis, resulting in a net increase of 46 votes toward the consensus diagnosis (Table S2). Diagnostic accuracy relative to the expert consensus diagnosis improved by 4% after genomics. For DPN‐M cases, diagnostic accuracy improved by 16% while in DPN/DPMT‐UMP cases it decreased by 1% (Figures 5 and 6). A McNemar test looking at paired nominal data resulted in a *p*‐value of 0.0001 with a confidence interval of 1.4 to 2.5, substantiating that the diagnostic shift in voting between Survey 1 and 2, which resulted in improved diagnostic accuracy, was not random.

**FIGURE 7:**
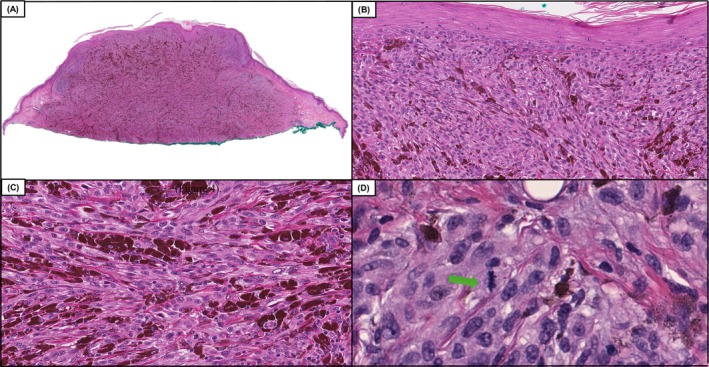
Case 2, a DPN‐M (deep penetrating nevus like melanoma) which resulted in distant metastasis with a genomic profile including variants *BRAF* V600E, *CTNNB1* S45F, *TERT* promoter c.‐124C>T, *NF1* L2073F, *SMARCA4* G1159K, and *GRIN2A* S556F and saw a 19% increase in diagnostic accuracy postgenomics. Low power demonstrates a compound highly cellular, but phenotypically heterogeneous melanocytic neoplasm expanding and filling the entire section (A, H&E, 50×). Intermediate power shows back‐to‐back nests of atypical epithelioid melanocytes with many intervening melanophages (B and C, H&E, 100× and 200×). Higher power demonstrates high‐grade nuclear atypia with easily identifiable mitotic figures denoted by a green arrow (3/mm^2^) (D, H&E, 400×).

Two DPN‐M patients developed clinically bulky metastatic melanoma to the lymph nodes. In case 2, prior to genomic data, 72% of votes favored a diagnosis of DPN‐M. After Survey 2 which demonstrated pathogenic variants in the following genes: *BRAF, CTNNB1, DNMT3A, GRIN2A, NF1, SMARCA4, and TERT‐p*, this increased to 91% (29 votes). This was a net improvement of 19% (six voters) in the diagnostic accuracy of recognizing this case as a DPN‐M (Figure 7).

**FIGURE 8:**
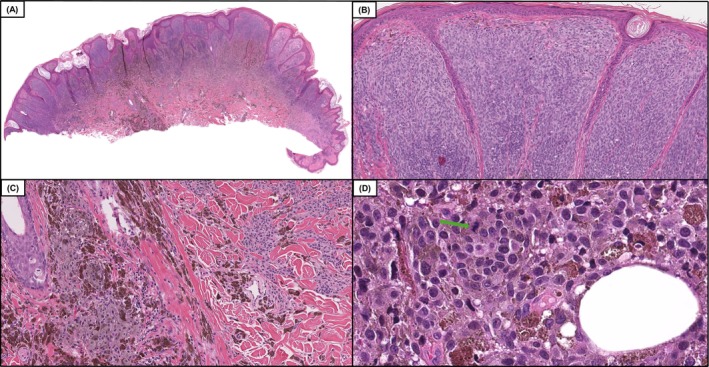
Case 39, a DPN‐M (deep penetrating nevus like melanoma) which resulted in distant metastasis, in which the diagnostic accuracy decreased by 6% following the inclusion of genomic data including *NRAS* Q61R, *CTNNB1* S37F, *CDKN2A* P48R, *ARID1B* P1314L, *NF2* P580L. Low power demonstrates an asymmetric compound melanocytic neoplasm with prominent phenotypic heterogeneity (A, H&E, 50×). Intermediate power shows expansile growth of atypical and mitotically active melanocytes, filling and expanding the papillary dermis, highly suggestive of a malignant melanocytic neoplasm (B, H&E, 100×). Intermediate power demonstrates a plexiform arrangement of pigmented epithelioid melanocytes typical of melanocytic neoplasms in the deep penetrating nevus family (C, H&E, 200×). High power magnification of the base of the lesion shows sheet‐like growth of severely atypical and mitotically active melanocytes (denoted by green arrow) with pigmented cytoplasm within the subcutis (D, H&E, 400×).

In the second DPN‐M patient with clinically bulky metastasis to the lymph node, case 39, 78% of voters favored a diagnosis of DPN‐M prior to genomics. After genomics which showed pathogenic alterations in the following genes: *NRAS*, *CTNNB1*, *NF2*, *CDKN2A*, and *ARID1B*, this decreased to 72%. There was a net decrease of two votes from DPN‐M to DPMT‐UMP (Figure 8).

## 4 Discussion

Despite most participants having limited experience with molecular diagnostics, we saw improvement in interobserver agreement and diagnostic accuracy after genomic data. Interobserver agreement improved from a Fleiss' Kappa of 0.41 to 0.51 in assessing a case as DPN‐M or not. Additionally, there was an overall improvement in diagnostic accuracy of 4%, primarily driven by a 16% increase in accurate diagnosis of DPN‐M cases.

The improved diagnostic accuracy in the DPN‐M cases mostly resulted from recognition of pathogenic variants in key tumor progression genes such as *TERT‐*promoter or *CDKN2A*. Four patients had positive SLNB and two developed clinically bulky metastatic melanoma to the lymph nodes. Since adjuvant therapy is now being considered for earlier stages of melanoma and has shown therapeutic benefits, accurate identification of these DPN‐M is significant.

The two DPN‐M cases resulting in bulky metastatic disease demonstrate how genomic data can improve accuracy as well as how it may be misinterpreted. In case 2, 72% of the diagnostic votes in Survey 1 were for melanoma (Figure 7). *TERT* promoter mutation was among the pathogenic variants included in Survey 2 after which votes for melanoma improved to 91%. In the second case (case 39), 78% of diagnostic votes were in favor of melanoma prior to genomics (Figure 8). After genomics, this decreased to 72%. We suspect that the absence of a *TERT* promoter mutation resulted in the net decrease in votes for melanoma. This case had pathogenic variants in *CDKN2A* and *ARID1B*. The literature suggests that pathogenic variants in *CDKN2A* would be unusual in a benign DPN [9, 11, 12].

While pathogenic variants in *CDKN2A* can be seen in some benign melanocytic neoplasms [13, 14], it's possible that a driver mutation in the MAP kinase pathway in conjunction with pathogenic variants in *CTNNB1* or *APC* and *CDKN2A* is sufficient for malignant transformation [11]. This is significant since pathogenic variants in *CDKN2A* were present in 4 of 11 DPN‐M cases (Table S1). Among pathogenic variants frequently seen in melanoma tumor progression, this was second only to pathogenic variants in *TERT‐*promoter (7 of 11 cases). Most participants recognized the importance of *TERT*‐promoter; however, many did not recognize the significance of pathogenic variants in *CDKN2A* in this diagnostic scenario. This emphasizes the need for more studies and education in interpreting genomic data. Pathogenic variants for *CDKN2A* were not identified in any of the nonmelanoma cases of this study though by IHC there were two nonmelanoma cases that had complete loss of P16. Further data is required to determine if the identification of pathogenic variants in *CDKN2A* by NGS has greater specificity for melanoma than complete loss of staining of P16 by IHC in this specific diagnostic scenario.

While not entirely diagnostic, the presence of multiple pathogenic variants in SWI/SNF genes was also more typical of DPN‐M than DPN [1, 9, 15, 16]. However, DPN‐M can occur without pathogenic variants involving any of these genes. Hence, if prior to genomics a diagnosis of melanoma is favored, one should not detract from that based on the absence of familiar tumor progression genes. If there is uncertainty and the genetics are unrevealing, a diagnosis of DPMT‐UMP may be reasonable. Furthermore, some benign DPNs may have a relatively high number of pathogenic variants involving less critical genes. Therefore, one should not endorse a melanoma diagnosis based purely on the number of pathogenic variants but rather interpret the data with greater nuance.

A limitation of this study is the modest size of the cohort (*n* = 39), as well as the utilization of two different NGS platforms. The PGDx and the Tempus xT/xO panels both have a variant allele frequency cutoff of 5% and similar coverage for genes of interest such as driver genes in the MAPkinase pathway, genes in the WNT pathway, genes in the SWI/SNF complex, *TERT*‐promoter and *CDKN2A*. Additionally, some of the metrics utilized such as percentage agreement can be impacted by convergence on an incorrect diagnosis. More studies are needed both to determine if the findings from a study environment will reflect real‐world experiences and whether the findings would be applicable to nonexpert pathologists. Finally, greater available clinical follow‐up would help further substantiate the results.

In summary, NGS studies are a powerful tool which can be exploited for diagnostic purposes. In this study, experts assessed a diagnostically challenging set of melanocytic tumors from the WNT‐activated family. Interobserver agreement and diagnostic accuracy in Survey 2, which included the genomic data, surpassed the values in Survey 1, which were based on morphology alone. The level of improvement may be considerably magnified with increased studies and educational efforts regarding the bioinformatics of these tumors.

## Funding

This work was supported by the IDP Foundation and with the support of the Greg and Anna Brown Foundation.

## Ethics Statement

This study was approved by our Institutional Review Board (IRB) (STU00001127). Given the retrospective chart review design, a waiver of HIPAA authorization was granted for research purposes.

## Consent

Informed consent was not required for this study.

## Conflicts of Interest

Dr. Pedram Gerami has received royalties for textbooks from Elsevier. Dr. Richard A. Scolyer has received fees for professional services from SkylineDx BV, IO Biotech ApS, MetaOptima Technology Inc., F. Hoffmann‐La Roche Ltd., Evaxion, Provectus Biopharmaceuticals Australia, Qbiotics, Novartis, Merck Sharp & Dohme, NeraCare, AMGEN Inc., Bristol‐Myers Squibb, Myriad Genetics, GlaxoSmithKline. The other authors declare no conflicts of interest.

## Supporting information


**Table S1:** Survey 2 containing clinical and genomic information for 39 cases.

**Table S2:** Distribution of votes by case and confusion matrix.

## Data Availability

The data that support the findings of this study are available from the corresponding author upon reasonable request.
