# Supplementary material for: The Impact of Next‐Generation Sequencing on Interobserver Agreement and Diagnostic Accuracy of Deep Penetrating Melanocytic Neoplasms
Source: J Cutan Pathol. 2025 Dec 23;53(3):293–301. doi: 10.1111/cup.70049 (PMC12867587; doi:10.1111/cup.70049)
Supplement: Supplementary file 2 — Table S2: Distribution of votes by case and confusion matrix. [file CUP-53-293-s002.docx]

|  | Case |  |  | | | | | | |  |
| --- | --- | --- | --- | --- | --- | --- | --- | --- | --- | --- |
|  |  | Consensus Vote | Votes for Melanoma | Votes for non-Melanoma |  |  | |  | |  |
|  | 1 | Melanoma | 9 | 23 |  |  | |  | |  |
|  |  |  | 22 | 10 |  |  | |  | |  |
|  | 2 | Melanoma | 23 | 9 |  |  | |  | |  |
|  |  |  | 29 | 3 |  |  | |  | |  |
|  | 3 | Non-Melanoma | 5 | 27 |  |  | |  | |  |
|  |  |  | 4 | 28 |  |  | |  | |  |
|  | 4 | Non-Melanoma | 3 | 29 |  |  | |  | |  |
|  |  |  | 0 | 32 |  |  | |  | |  |
|  | 5 | Non-Melanoma | 16 | 16 |  |  | |  | |  |
|  |  |  | 6 | 26 |  |  | |  | |  |
|  | 6 | Non-Melanoma | 0 | 32 |  |  | |  | |  |
|  |  |  | 1 | 31 |  |  | |  | |  |
|  | 7 | Non-Melanoma | 0 | 32 |  |  | |  | |  |
|  |  |  | 1 | 31 |  |  | |  | |  |
|  | 8 | Non-Melanoma | 1 | 31 |  |  | |  | |  |
|  |  |  | 1 | 31 |  |  | |  | |  |
|  | 9 | Non-Melanoma | 1 | 31 |  |  | |  | |  |
|  |  |  | 0 | 32 |  |  | |  | |  |
|  | 10 | Non-Melanoma | 0 | 32 |  |  | |  | |  |
|  |  |  | 4 | 28 |  |  | |  | |  |
|  | 11 | Non-Melanoma | 5 | 27 |  |  | |  | |  |
|  |  |  | 1 | 31 |  |  | |  | |  |
|  | 12 | Non-Melanoma | 4 | 28 |  |  | |  | |  |
|  |  |  | 0 | 32 |  |  | |  | |  |
|  | 13 | Non-Melanoma | 1 | 31 |  |  | |  | |  |
|  |  |  | 2 | 30 |  |  | |  | |  |
|  | 14 | Non-Melanoma | 10 | 22 |  |  | |  | |  |
|  |  |  | 12 | 20 |  |  | |  | |  |
|  | 15 | Melanoma | 29 | 3 |  |  | |  | |  |
|  |  |  | 31 | 1 |  |  | |  | |  |
|  | 16 | Non-Melanoma | 1 | 31 |  |  | |  | |  |
|  |  |  | 1 | 31 |  |  | |  | |  |
|  | 17 | Non-Melanoma | 3 | 29 |  |  | |  | |  |
|  |  |  | 0 | 32 |  |  | |  | |  |
|  | 18 | Non-Melanoma | 5 | 27 |  |  | |  | |  |
|  |  |  | 4 | 28 |  |  | |  | |  |
|  | 19 | Non-Melanoma | 2 | 30 |  |  | |  | |  |
|  |  |  | 10 | 22 |  |  | |  | |  |
|  | 20 | Non-Melanoma | 2 | 30 |  |  | |  | |  |
|  |  |  | 0 | 32 |  |  | |  | |  |
|  | 21 | Non-Melanoma | 3 | 29 |  |  | |  | |  |
|  |  |  | 3 | 29 |  |  | |  | |  |
|  | 22 | Melanoma | 23 | 9 |  |  | |  | |  |
|  |  |  | 31 | 1 |  |  | |  | |  |
|  | 23 | Melanoma | 21 | 11 |  |  | |  | |  |
|  |  |  | 14 | 18 |  |  | |  | |  |
|  | 24 | Melanoma | 27 | 5 |  |  | |  | |  |
|  |  |  | 30 | 2 |  |  | |  | |  |
|  | 25 | Non-Melanoma | 4 | 28 |  |  | |  | |  |
|  |  |  | 11 | 21 |  |  | |  | |  |
|  | 26 | Non-Melanoma | 0 | 32 |  |  | |  | |  |
|  |  |  | 0 | 32 |  |  | |  | |  |
|  | 27 | Non-Melanoma | 1 | 31 |  |  | |  | |  |
|  |  |  | 1 | 31 |  |  | |  | |  |
|  | 28 | Non-Melanoma | 1 | 31 |  |  | |  | |  |
|  |  |  | 0 | 32 |  |  | |  | |  |
|  | 29 | Non-Melanoma | 3 | 29 |  |  | |  | |  |
|  |  |  | 2 | 30 |  |  | |  | |  |
|  | 30 | Non-Melanoma | 0 | 32 |  |  | |  | |  |
|  |  |  | 1 | 31 |  |  | |  | |  |
|  | 31 | Melanoma | 15 | 17 |  |  | |  | |  |
|  |  |  | 15 | 17 |  |  | |  | |  |
|  | 32 | Melanoma | 13 | 19 |  |  | |  | |  |
|  |  |  | 28 | 4 |  |  | |  | |  |
|  | 33 | Non-Melanoma | 15 | 17 |  |  | |  | |  |
|  |  |  | 20 | 12 |  |  | |  | |  |
|  | 34 | Melanoma | 2 | 30 |  |  | |  | |  |
|  |  |  | 23 | 9 |  |  | |  | |  |
|  | 35 | Non-Melanoma | 4 | 28 |  |  | |  | |  |
|  |  |  | 0 | 32 |  |  | |  | |  |
|  | 36 | Non-Melanoma | 1 | 31 |  |  | |  | |  |
|  |  |  | 1 | 31 |  |  | |  | |  |
|  | 37 | Non-Melanoma | 0 | 32 |  |  | |  | |  |
|  |  |  | 5 | 27 |  |  | |  | |  |
|  | 38 | Melanoma | 14 | 18 |  |  | |  | |  |
|  |  |  | 11 | 21 |  |  | |  | |  |
|  | 39 | Melanoma | 25 | 7 |  |  | |  | |  |
|  |  |  | 23 | 9 |  |  | |  | |  |
|  |  |  |  |  |  |  | |  | |  |
|  |  |  | Survey 1 |  |  |  | |  | |  |
|  |  |  | Survey 2 |  |  |  | |  | |  |
|  |  |  |  |  |  | |  | |  | |
|  |  |  |  |  |  | |  | |  | |
|  | Survey 1 |  | Predicted Votes |  |  | |  | |  | |
|  |  |  | false positive | false negative | Accuracy | | 0.806891 | |  | |
|  | Actual Votes | true positive | 202 | 91 | Precision | | 0.573864 | |  | |
|  |  | true negative | 150 | 805 | Recall | | 0.68942 | |  | |
|  |  |  |  |  |  | |  | |  | |
|  |  |  |  |  |  | |  | |  | |
|  | Survey 2 |  | Predicted Votes |  |  | |  | |  | |
|  |  |  | false positive | false negative | Accuracy | | 0.852564 | |  | |
|  | Actual Votes | true positive | 259 | 90 | Precision | | 0.733711 | |  | |
|  |  | true negative | 94 | 805 | Recall | | 0.74212 | |  | |
|  |  |  |  |  |  | |  | |  | |
|  |  |  |  |  |  | |  | |  | |
|  |  |  |  |  |  | |  | |  | |
|  |  |  |  |  |  | |  | |  | |
|  |  |  |  |  |  | |  | |  | |
|  |  |  |  |  |  | |  | |  | |
|  |  |  |  |  |  | |  | |  | |
|  |  |  |  |  |  | |  | |  | |
|  |  |  |  |  |  | |  | |  | |
|  |  |  |  |  |  | |  | |  | |
|  |  |  |  |  |  | |  | |  | |
|  |  |  |  |  |  | |  | |  | |
|  |  |  |  |  |  | |  | |  | |
|  |  |  |  |  |  | |  | |  | |
